# Calcinosis Cutis in Juvenile Systemic Sclerosis

**DOI:** 10.7759/cureus.59729

**Published:** 2024-05-06

**Authors:** Dhanush Balaji, Kavitha Mohanasundaram, Karpaka Vinayakam Gopalakrishnan

**Affiliations:** 1 Internal Medicine, Saveetha Medical College and Hospital, Saveetha Institute of Medical and Technical Sciences, Kanchipuram, IND; 2 General Medicine, Saveetha Medical College and Hospital, Saveetha Institute of Medical and Technical Sciences, Kanchipuram, IND

**Keywords:** systemic sclerosis interstitial lung disease(ssc-ild), auto immune rheumatological conditions, raynauds phenomenon, scleroderma, juvenile systemic sclerosis

## Abstract

Juvenile systemic sclerosis (JSSc) is a rare autoimmune disorder that primarily affects children and adolescents. It is thought to be caused by a confluence of immunological, environmental, and genetic variables. The disease is characterized by excessive collagen production. It can result in symptoms such as shortness of breath, chest pain, difficulty swallowing, high blood pressure, and kidney problems. Although calcinosis cutis is common in systemic sclerosis, it is very rare in JSSc. We report the case of a 14-year-old female who presented with complaints of breathlessness for four days and multiple lesions in the sacral region for two months. She underwent surgical excision for calcinosis cutis in dependent regions. Early diagnosis and treatment of the condition are of immense importance in preventing mortality.

## Introduction

Juvenile systemic sclerosis (JSSc), also known as juvenile systemic scleroderma, is a rare autoimmune disorder that primarily affects children and adolescents. It is a chronic condition characterized by abnormal thickening and hardening of the skin, as well as involvement of various internal organs. It is a rare disease with an estimated prevalence of 0.27-2.9 cases per million [1,2]. The mean age of onset of JSSc is 8-11 years. It is estimated that less than 10% of all patients develop JSSc before the age of 20. While the exact cause of JSSc is unknown, it is believed to involve a combination of genetic, environmental, and immune factors [3]. One of the hallmarks of JSSc is skin involvement. The skin becomes thickened and tight, especially over the face, hands, and feet. This can lead to joint stiffness and a limited range of motion. In addition to skin changes, JSSc can also affect internal organs such as the lungs, heart, kidneys, and gastrointestinal tract. This can result in symptoms such as shortness of breath, chest pain, difficulty swallowing, high blood pressure, and kidney problems. Living with JSSc can have a significant impact on a child’s daily life and overall well-being. Approximately 80% of people with systemic sclerosis develop lung fibrosis; approximately one-fourth develop progressive interstitial lung disease (ILD), with a 10-year mortality rate of around 40%, making it one of the leading causes of morbidity and mortality [3,4]. It is also important for affected children and their families to have a strong support network and access to specialized medical care. Ongoing education about the disease and its management can empower patients and their families to actively participate in their own care. We present a case of JSSc presenting with refractory calcinosis cutis and ILD.

## Case presentation

A 14-year-old female presented with complaints of breathlessness for four days and multiple lesions in the sacral region for over two months, with similar complaints in the past. She was also complaining of difficulty opening the mouth (microstomia) and discoloration of the fingers upon exposure to cold (monophasic Raynaud’s phenomenon, as only pallor was seen). It was determined that the dyspnea was mMRC Grade 1, and it had also gotten worse over the night. There was no history of fever, chest pain, hemoptysis, palpitations, or any gastrointestinal symptoms. There was also a similar episode of breathlessness seven months ago, for which she received treatment at a local clinic. There was no family history of similar complaints.

**Figure 1:**
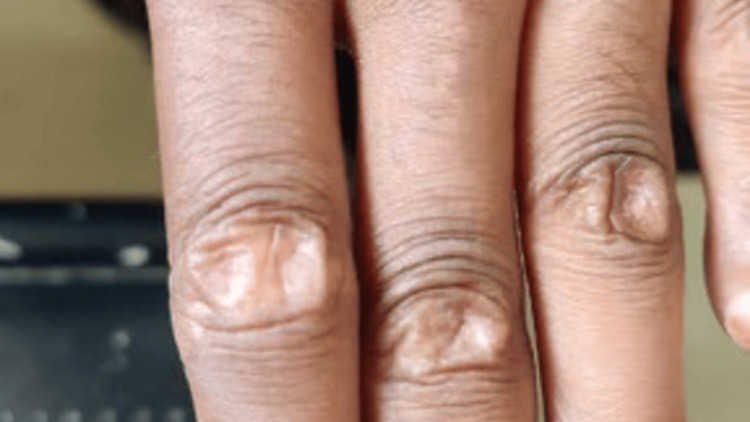
The image of the digits shows hypopigmented skin lesions associated with palpable nodules consistent with calcinosis cutis.

**Figure 2:**
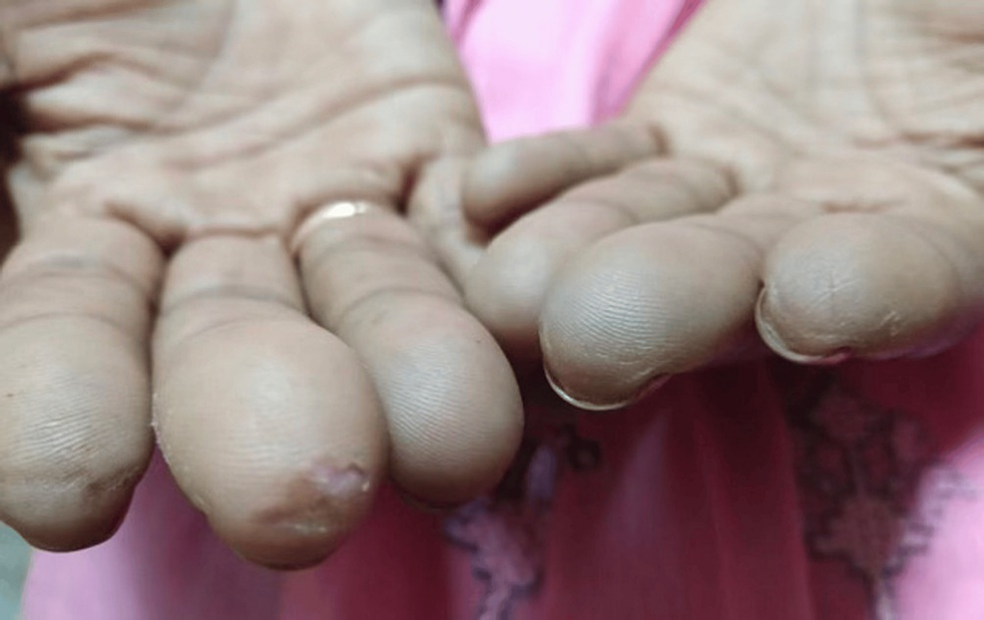
The image shows the presence of healed digital tip ulcers, which can occur as a result of reduced blood flow due to the narrowing of the vessels.

Upon examination, there was a presence of pseudo-clubbing; there was also thickening and tightening of the skin involving the face, neck, bilateral upper limb, and lower limb, causing flexion deformity of the fingers (Figure 1), extending beyond the elbow and knee, respectively. Salt and pepper pigmentation was seen in the neck region. Healed digital pitted scars were observed as well (Figure 2). In the face, there was thinning of the lips, pursed lips, a pinched nose, and a rather characteristic masked face. Calcinosis cutis was observed in the gluteal and bilateral elbow regions, and Gottron papules were present over the knuckles. There was no muscle weakness. Telangiectasias were not observed. Also, bilateral crepitations were heard on auscultation.

Investigations

**Figure 3:**
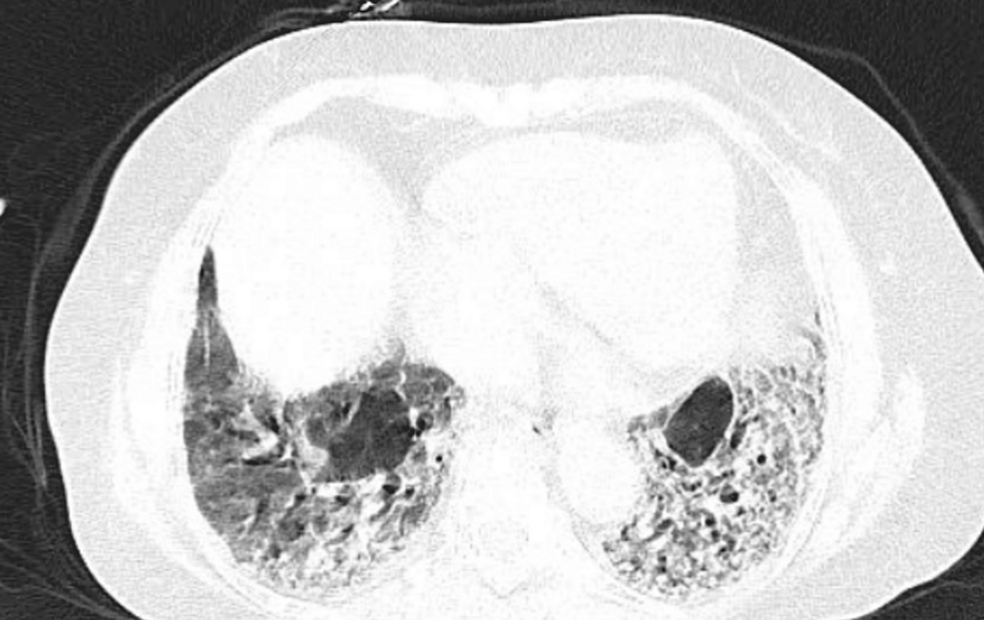
This is a thoracic HRCT that showed several “honeycomb” lesions in both lower lobes. Additionally, there were two characteristics that are indicative of subsequent ILD: intralobular septal thickness and glasses haziness. HRCT, high-resolution computed tomography; ILD, interstitial lung disease

The hemoglobin level was found to be 12.8 g/dl. The total white blood cell count was 5,600 cells. The total platelet count was 3.35 lakhs/mm. The erythrocyte sedimentation rate and C-reactive protein were found to be 52 mm/h and <5 mg/dl, respectively. The inflammatory markers were found to be elevated, and their values represented non-specific inflammation in the body. Also, creatine kinase and urine albumin were measured to assess renal function, and they were found to be normal. The presence of antinuclear antibodies was checked by immunofluorescent antibodies. There was the presence of granular nucleoplasm with distinct nucleoli, and with a titer of 1:160, the presence of the anti-Scl 30+ antibody was confirmed. To further investigate the cause of breathlessness, a high-resolution computed tomography of the thorax was performed, which revealed the presence of multiple “honeycomb” lesions in both the lower lobes. There was also the presence of glass haziness and intralobular septal thickening, and these features are suggestive of secondary ILD (Figure 3).

An X-ray of the bilateral foot was done, and the presence of fluffy soft tissue calcification was observed, which is suggestive of calcinosis cutis. The ejection fraction was found to be 64%, which is normal, and there was no evidence of pulmonary artery hypertension. The diffusing capacity for the carbon monoxide test was done and found to be less than 80%; a 6-minute walk test was also normal. The modified Rodnan score was found to be 27, which measures skin thickness, and the JSSc severity score was found to be 14, which predicts the severity of the involvement of various organs. The pulmonary function test showed a forced expiratory volume at 1 second (FEV1) of 57% and a forced vital capacity (FVC) of 47%. FEV1/FVC = 0.83, indicative of a restrictive pattern of lung disease.

Treatment

The patient was treated with a tablet of mycophenolate mofetil dosage of 500 mg twice a day and a tablet of prednisolone dosage of 5 mg once a day, which was to be consumed orally. She was also given a calcium channel blocker (CCB) to treat Raynaud’s phenomenon. Tretinoin 0.05% cream was given for topical application. To treat ILD, budamate 400 mg aerosol therapy along with tablet doxophylline 200 mg was prescribed to be used twice daily. Pantoprazole 40 mg was given once a day. Bisphosphonates were also prescribed, which will help prevent further calcium deposition, thereby preventing calcinosis cutis. Since the calcinosis cutis did not show any major improvement, she underwent surgical excision for calcinosis cutis in dependent regions, and phototherapy was planned post-surgery. After surgical excision for calcinosis cutis in dependent regions, the patient’s response and recovery were closely monitored, and her symptoms of breathlessness and multiple swellings over the gluteal region were assessed for improvement. The patient was followed up for six months post-surgery, during which her complaints of breathlessness improved, she recovered well from the ILD, and she had a great response to treatment.

## Discussion

JSSc, also known as juvenile scleroderma, is a chronic autoimmune disorder characterized by excessive collagen production. It primarily affects the skin but can also impact the blood vessels, organs, and joints. The early stages of JSSc often present with localized symptoms, such as hardening and tightening of the skin, particularly in the hands, face, and limbs. As the disease progresses, it can involve internal organs, leading to widespread complications. These complications may include pulmonary hypertension, kidney problems, gastrointestinal issues, and joint stiffness [5]. There are a variety of factors that affect the prognosis. This can include organ involvement, time of diagnosis, and whether the systemic sclerosis is diffuse or limited. All of these factors lead to a poor prognosis [6,7]. The early diagnosis and treatment of the condition are of immense importance. In most cases, the diagnosis is based on clinical presentations and immunological workups. Testing the presence of antinuclear antibodies by immunofluorescence assay remains the standard practice, but nail capillaroscopy can also be standardized as it can allow us to differentiate between primary and secondary Raynaud’s phenomenon [8]. Oral video capillaroscopy can be developed into a reliable technique for detecting oral microcirculation abnormalities [9]. The 2013 American College of Rheumatology (ACR)/European League Against Rheumatism (EULAR) classification criteria for systemic sclerosis still maintains its effectiveness in diagnosis and monitoring disease prognosis [10-13]. This patient was started on the tablet nifedipine (60 mg), a CCB, to treat the monophasic Raynaud’s phenomenon. She was also started on sildenafil (20 mg) twice a day for the same purpose. Since it is an autoimmune condition, she was given immunosuppressants such as steroids (prednisolone) and disease-modifying antirheumatic drugs (mycophenolate mofetil). She was also given nintedanib in order to treat her lung fibrosis (ILD). Nintedanib is a VEGF, BDGF, and FGR inhibitor that can be used as an anti-fibrosing agent. In order to treat any renal complications, ACE inhibitors like enalapril can be given. Similarly, to treat pulmonary arterial hypertension, bosentan, a PDE-5 inhibitor, can be given. Advances in genetic research will allow us to identify potential genetic markers associated with the disease. Additionally, studies exploring the role of the immune system and environmental factors can shed light on the underlying mechanisms of JSSc [14-16]. Clinical trials can also be done, which can help investigate new medications and therapies aimed at managing symptoms and preventing complications. These advancements can offer hope for improved outcomes and quality of life for individuals living with JSSc.

## Conclusions

JSSc, as such, is an orphan disease. Its association with calcinosis cutis, and ILD is even rarer. It can be associated with various complications like ILD, renal crises, pulmonary arterial hypertension, and Raynaud’s phenomenon, among others, which can become life-threatening. Thus, early detection and prompt management are needed to prevent morbidity and mortality.
